# Lessons on brain edema in HE: from cellular to animal models and clinical studies

**DOI:** 10.1007/s11011-023-01269-5

**Published:** 2023-08-22

**Authors:** Katarzyna Pierzchala, Anna Hadjihambi, Jessie Mosso, Rajiv Jalan, Christopher F. Rose, Cristina Cudalbu

**Affiliations:** 1 CIBM Center for Biomedical Imaging, Lausanne, Switzerland; 2 Animal Imaging and Technology, EPFL, Lausanne, Switzerland; 3 The Roger Williams Institute of Hepatology London, Foundation for Liver Research, London, SE5 9NT UK; 4 Faculty of Life Sciences and Medicine, King’s College London, London, UK; 5 Laboratory for Functional and Metabolic Imaging (LIFMET), EPFL, Lausanne, Switzerland; 6 Liver Failure Group, Institute for Liver and Digestive Health, University College London, Royal Free Campus, London, UK; 7 European Foundation for the Study of Chronic Liver Failure (EF Clif), Barcelona, Spain; 8 Hépato-Neuro Laboratory, Centre de Recherche du Centre Hospitalier de l’, Université de Montréal (CRCHUM), Montreal, QC H2X 0A9 Canada; 9 Department of Medicine, Faculty of Medicine, Université de Montréal, QC Montreal, H3T 1J4 Canada

**Keywords:** Brain edema, Brain water content, Hepatic encephalopathy, Type A hepatic encephalopathy, Type C hepatic encephalopathy, Astrocytes, Neurons, Swelling, *In-vivo* and *ex-vivo* measurements

## Abstract

Brain edema is considered as a common feature associated with hepatic encephalopathy (HE). However, its central role as cause or consequence of HE and its implication in the development of the neurological alterations linked to HE are still under debate. It is now well accepted that type A and type C HE are biologically and clinically different, leading to different manifestations of brain edema. As a result, the findings on brain edema/swelling in type C HE are variable and sometimes controversial. In the light of the changing natural history of liver disease, better description of the clinical trajectory of cirrhosis and understanding of molecular mechanisms of HE, and the role of brain edema as a central component in the pathogenesis of HE is revisited in the current review. Furthermore, this review highlights the main techniques to measure brain edema and their advantages/disadvantages together with an in-depth description of the main *ex-vivo/in-vivo* findings using cell cultures, animal models and humans with HE. These findings are instrumental in elucidating the role of brain edema in HE and also in designing new multimodal studies by performing *in-vivo* combined with *ex-vivo* experiments for a better characterization of brain edema longitudinally and of its role in HE, especially in type C HE where water content changes are small.

## Introduction

Brain edema is considered as a common feature associated with hepatic encephalopathy (HE). However, its central role as cause or consequence of HE, as well as its implication in the development of the neurological alterations linked to HE are still under debate. Moreover, it is still unclear whether HE and brain edema are the manifestations of the same pathophysiological mechanism or two different cerebral manifestations of brain dysfunction in liver disease, especially in the context of type C (cirrhosis) HE (Bosoi and Rose 2013; Cudalbu and Taylor-Robinson 2019). It is now well accepted that type A (acute liver failure (ALF)) and type C HE are biologically and clinically different, leading to different manifestations of brain edema (Bosoi and Rose 2013). For instance, in type C HE, the levels of ammonia in the blood are lower, variable and there is sufficient time for effective compensation and stabilization of the osmolyte shift to counteract the osmotic imbalance induced by the astrocytic accumulation of glutamine (Gln). As a result, the findings on net brain edema and type of edema in type C HE are variable and sometimes controversial (Cudalbu and Taylor-Robinson 2019). On the other hand, in type A HE, the natural history of the syndrome is rapid with higher ammonia levels in the blood, thus not allowing the system to compensate for metabolic changes.

As such, the role of brain edema as a central component in the pathogenesis of HE should be revisited in the light of the changing natural history of liver disease, better description of the clinical trajectory of cirrhosis and understanding of molecular pathogenesis of HE (Rose et al. 2020). The role of brain edema as the central mechanism of HE was driven by two observations. First, the seminal observation that astrocytes expressed glutamine synthase (GS), and that the production of Gln would lead to cytosolic hypertonicity and astrocytes would swell (Martinez-Hernandez et al. 1977). Second, the first description of a swollen astrocyte in post-mortem brain biopsies from patients dying due to acute liver failure (Type A HE) (Kato et al. 1992). These early findings should be examined in the light of subsequent developments in our understanding of the pathobiology of HE and its clinical manifestations.

Brain edema represents a net accumulation of fluid, mainly water, in the intracellular or extracellular spaces (ECS) of the brain, occurring on the background of an osmotic gradient. Cerebral edema is commonly observed in a variety of brain injuries. It can appear due to cytotoxic (alterations in cellular metabolism causing accumulation of osmotic molecules followed by entry of water to re-establish the osmotic equilibrium; and sometimes an increase in blood-brain-barrier (BBB) permeability) and/or vasogenic (physical breakdown of BBB) mechanisms (Bosoi and Rose 2013). It is important to emphasize that it is unusual for only vasogenic or cytotoxic edema to exist in isolation, therefore labeling a particular case of edema as “vasogenic” or “cytotoxic” cannot be rigidly applied. Overall, the development of one type of edema will gradually lead to the development of the other type, which also seems to be the case in HE where both types of edema might coexist, thus sometimes rendering it difficult to clearly distinguish them. It is important to mention that the terminology used is sometimes misleading, as edema should always refer to a net increase in water content in the brain. Sometimes edema is also referred to as a shift of water from one compartment to the other without a net increase of water amount in the brain, even though it is suspected that such a shift would always be accompanied by some degree of net brain edema through other mechanisms, such as osmotic gradient across the BBB (Bosoi and Rose 2013).

### Role of astrocyte swelling

Although many lines of investigation over the years have confirmed the pathological role of astrocytes in animal models and humans of severe hyperammonemia and liver failure, it has also become clear that additional cell types such as the endothelial cells, neurons, microglia and pericytes in the brain are also involved in the pathogenesis of HE (Flatt et al. 2021; Hadjihambi et al. 2022; Haussinger et al. 1994, 2022; Mosso et al. 2022b; Pelle et al. 2022; Pierzchala et al. 2022; Rackayova et al. 2020, 2021; Rose et al. 2020; Simicic et al. 2022). Subsequent investigations have indicated that the hypothesis of ammonia-Gln-brain swelling is perhaps too simplistic. Indeed, mechanisms are much more complex and clinical observations, imaging studies and postmortem brain biopsies from patients dying with HE have indicated evidence of structural brain injury, presence of neuroinflammation, altered brain metabolism with Gln increase and gene expression affecting multiple pathways, endothelial dysfunction, alterations of blood and glymphatic flow, altered cellular bioenergetics, deposition of metals, mitochondrial dysfunction, lactate transport and senescence (Braissant et al. 2019; Haussinger et al. 2022; Pierzchala et al. 2022; Rose et al. 2020; Simicic et al. 2022). The most important learning from these advances is the associated clinical implications. An example is the observation that in a proportion of patients with HE who undergo liver transplantation, recovery from HE is incomplete, whist the brain continues to show loss of volume, arguing that HE is associated with neuronal loss (Garcia-Martinez et al. 2011; Jalan and Rose 2022; Ochoa-Sanchez et al. 2021). Another example is the lack of a direct correlation between therapies such as lactulose and rifaximin that are routinely used in clinical practice and either ammonia levels or brain swelling (Haussinger et al. 2022; Rose et al. 2020).

### Clinical relevance of cerebral edema in HE

In reviewing the paper by Kato et al. (1992), in addition to demonstrating astrocyte swelling in type A HE, their main observations were regarding endothelial cell vacuolization, enlargement and vacuolation of the basement membrane and expansion of the extracellular processes. Derangements of the pericytes were also observed. Although they suggested that cytotoxic edema may be operative, they were not convinced that this was not due to post-mortem effects. It appears that what they were describing was indeed vasogenic brain edema, loss of pericytes, mitochondrial disruption and loss of integrity of the BBB. It is tempting to hypothesize that these early findings might have driven the studies that followed (Martinez-Hernandez et al. 1977) to support the ammonia-Gln-brain swelling hypothesis and partly overlooked the other observations made. Indeed, subsequent studies went on to show the importance of alterations in cerebral blood flow (CBF) (both hyperemia and ischemia) (Jalan et al. 2004; Wendon et al. 1994), systemic and neuroinflammation (Rolando et al. 2000) and, evidence that in the sickest patients with ALF with uncontrolled intracranial hypertension the BBB breaks down and the brain becomes a net cytokine producer (Wright et al. 2007b). Further experimental studies in animal models of ALF showed that intracranial pressure (ICP) could be modulated using albumin dialysis without any impact on the ammonia-Gln-brain swelling pathway, arguing for the limited involvement of cytotoxic edema in the pathogenesis of HE in ALF (Sen et al. 2006).

The link to cerebral edema being the central operative mechanism of HE in cirrhosis is challenging as type C HE develops differently from type A HE and presents lower blood ammonia levels and water content increase, with a longer disease time course. Moreover, these patients show a very wide spectrum of clinical and pathophysiological phenotypes that manifest with varying severities of HE (Rose et al. 2020). It is difficult to conceive how cerebral edema can be pathogenetically important in mobile patients with minimal or Grade 1 HE. Nonetheless these patients show an increased brain Gln and decreased brain osmolytes, signs of osmotic stress which cannot be neglected and which were interpreted as low-grade edema as in type C HE (Haussinger 2006; Haussinger et al. 1994). Indeed, although some of the early imaging studies have measured increased brain Gln and altered brain water, the relationship between one and the other is still not fully elucidated (Cudalbu and Taylor-Robinson 2019). Several studies using magnetic resonance imaging (MRI) and positron emission tomography (PET) scanning have pointed to additional features such as deposition of metal, altered CBF, bioenergetics, oxygen consumption and evidence of neuroinflammation (Haussinger 2006; Haussinger et al. 1994; Mosso et al. 2022b). In patients with Grades 2–4 HE, cerebral edema is observed (Oeltzschner et al. 2016; Shah et al. 2008; Winterdahl et al. 2019). This is unlikely to be due to cytotoxic brain edema alone and more likely it is attributed to multiple related factors, such as neuroinflammation, altered metabolism and CBF and neuronal injury. Studies in animal models of type C HE have, however, showed an increase in brain water content (Bosoi et al. 2012), Gln, decrease of brain osmolytes and structural changes in astrocytes, neurons and microglia (Bosoi et al. 2014; Braissant et al. 2019; Jaeger et al. 2019; Pierzchala et al. 2022; Rackayova et al. 2016, 2020, 2021; Rose 2010; Simicic et al. 2022). An increase in brain Gln will eventually lead to cellular microstructural changes despite the osmoregulation (i.e. release of other brain osmolytes) (Mosso et al. 2021, 2022a). Whether this is involved in patients with covert HE or overt HE remains to be investigated. Therefore, additional longitudinal studies are needed to follow brain Gln, water, microstructural changes, oxidative stress (OS), neuroinflammation, bioenergetics, metabolism together with blood ammonia changes in patients with type C HE.

Acute-on-chronic liver failure (ACLF) is a syndrome that occurs in patients with cirrhosis who are hospitalized with acute decompensation. It is characterized by the failure of hepatic and extrahepatic organs and high rates of mortality (Arroyo et al. 2020). The syndrome is characterized pathophysiologically by systemic inflammation (Claria et al. 2016) consequent on accumulation of pathogen and damage associated molecular patterns, as well as altered signaling that culminates in mitochondrial dysfunction (Moreau et al. 2021). The occurrence of HE in patients with ACLF significantly increases the risk of death (Cordoba et al. 2014). From the pathophysiologic perspective, cerebral edema is observed in about 5% of patients (Joshi et al. 2014). In animal models of ACLF, cerebral edema is frequent and is associated with hyperammonemia, neuroinflammation, altered brain oxygenation, neuronal loss and disturbances of the BBB (Rose et al. 2020; Sawhney et al. 2016). The severity of hyperammonemia is an independent predictor of mortality and a reduction in the ammonia levels is associated with improved survival. Taken together, the brain dysfunction of ACLF is clinically, pathophysiologically and prognostically distinct from both types A and C HE. At present, no special category has been allocated to HE in ACLF patients and continues to be classified under Type C HE (Jalan and Rose 2022). This issue should be revisited in future consensus discussions.

**Fig. 1:**
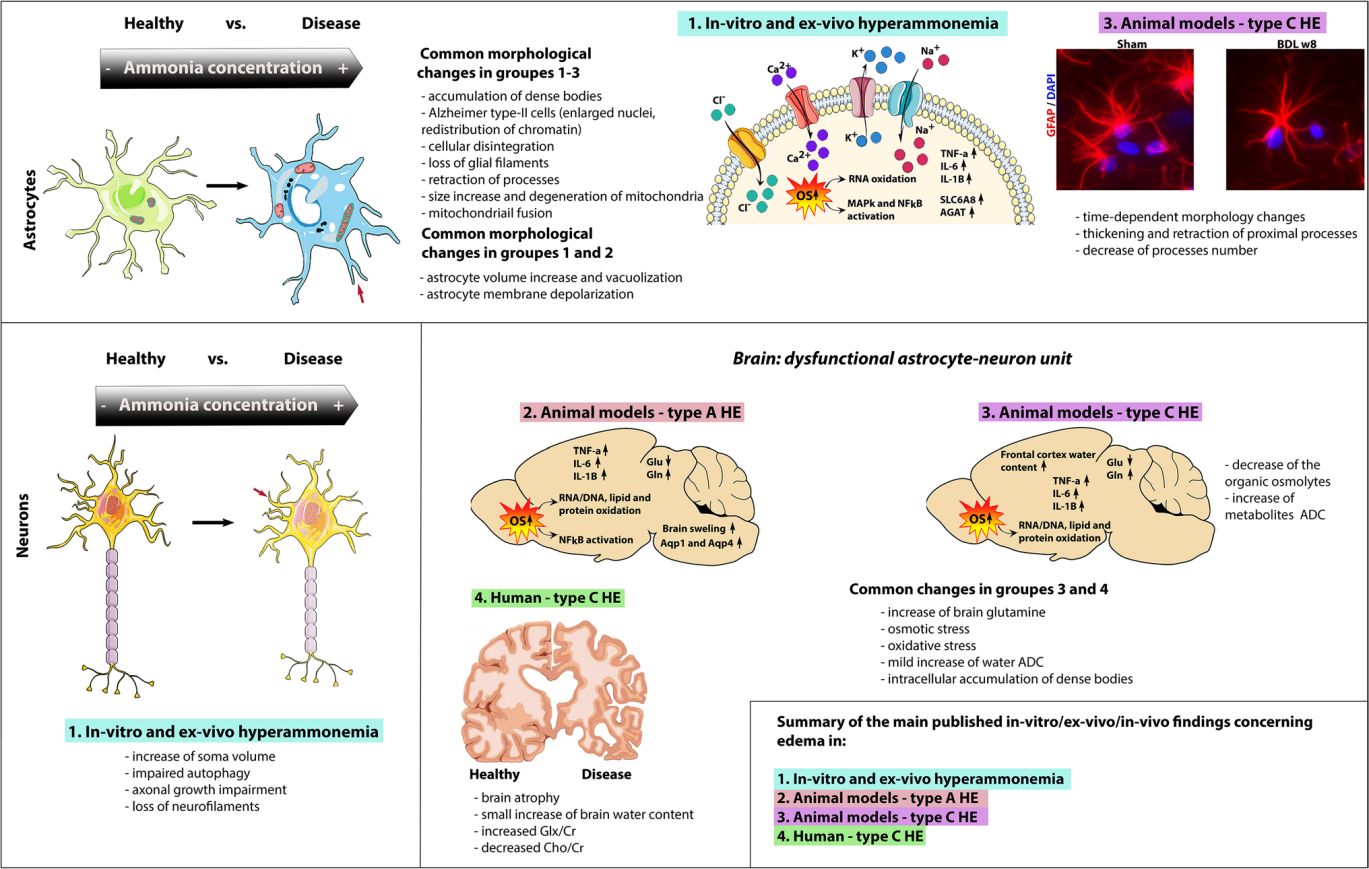
Summary of the current knowledge related to edema formation from *in-vitro*, *ex-vivo* brain tissue, and *in-vivo* studies in different HE animal models and patients

Of note, the current review is not questioning the presence of brain edema in HE. However it is highlighting the fact that it is now accepted that HE is a multifactorial disease where brain edema might not be the only main event. Therefore, we believe that the role of cytotoxic astrocytic brain edema as being central to the pathogenesis of HE should be revisited considering the current understanding of the syndrome (Fig. 1).

## Current technics to measure brain edema

 Several *ex-vivo*/invasive or *in-vivo*/non-invasive methods have been used in the past to assess brain water content and consequently brain edema in both animal models and patients with HE. A detailed description of these methods can be found elsewhere (Cudalbu and Taylor-Robinson 2019). Table 1 presents a summary of the advantages and disadvantages of these methods, while the following tables (Tables 2, 3 and 4) show the main results obtained using these techniques in cell cultures, as well as different animal models and patients with HE (summarized in Fig. 1).

**Table 1:** Summary of the main methods used to investigate brain edema with their advantages and disadvantages highlighted

Method	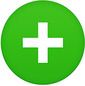	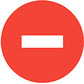
Gravimetry	+ allows a direct assessment of brain water content + quantitative + small samples can be used + easy to implement	- *ex-vivo* method, no longitudinal studies on the same animals possible - non-specific, does not allow an assessment at cellular level - no information on the type of edema
Dry/wet weight	+ allows a direct assessment of brain water content + estimate % changes, quantitative + fast method accessible to everyone	- *ex-vivo* method using freshly dissected tissue, no longitudinal studies on the same animals possible - non-specific, does not allow an assessment at cellular level - no information on the type of edema - precision can be sometimes a limiting factor in small sample size
Electron microscopy (EM)	+ extracellular space images with nanoscale resolution	- *ex-vivo* method - no longitudinal studies on the same animals possible - requires tissue fixation
Microscopy Live tissue super resolution 3D-STED and SUSHI	+ live-tissue real time imaging + allows visualization of the extracellular space (ECS) using fluorescent dyes + estimation of ECS volume fraction + astrocytes volume remodeling under osmotic stress	- *ex-vivo* method using freshly dissected tissue, no longitudinal studies on the same animals possible
Water mapping using MRI	+ allows a direct assessment of brain water content + estimate % changes, quantitative + good resolution allowing the investigation in all brain regions + non-invasive and allows *in-vivo* and longitudinal studies in the same animal + provides insights into the chronology of events involved in HE + translational	- non-specific, does not allow an assessment at cellular level - no information on the type of edema
Diffusion weighted magnetic resonance imaging (DW-MRI)	+ probes tissue microstructure at micrometer scale: changes in extra- and intra-cellular water diffusivity and fraction, in soma and processes radius, in orientation dispersion of fiber bundle + changes in the apparent diffusion coefficient can be linked to the type of edema (cytotoxic edema associated to a decreased apparent diffusion coefficient (ADC), vasogenic edema associated to an increased ADC) provided that the results are validated against other *ex-vivo* techniques as done in stroke + quantitative, translational + non-invasive and allows i*n-vivo *and longitudinal studies in the same animal + high sensitivity of water MR signal + provides insights into the chronology of events involved in HE	- indirect measurement: requires modelling of water diffusion properties - non-cell-specific - multiple interpretations possible - no direct assessment of brain water content
Brain volumetry using high resolution MRI data	+ allows a direct assessment of brain volumes + estimate % changes, quantitative + good resolution allowing the investigation in all brain regions + non-invasive and allows -*in-vivo* and longitudinal studies in the same animal + provides insights into the chronology of events involved in HE + translational	- no direct assessment of brain water content - non-specific, does not allow an assessment at cellular level - no information on the type of edema
Magnetization transfer MR imaging (MT) and FLAIR MR imaging	+ good resolution allowing the investigation in all brain regions + non-invasive and allows *in-vivo* and longitudinal studies in the same animal + provides insights into the chronology of events involved in HE + translational	- no direct assessment of brain water content - non-specific, does not allow an assessment at cellular level - no information on the type of edema
Magnetic Resonance Spectroscopy	+ probes brain metabolism directly in a quantitative manner + brain osmolytes are quantified thus allowing to directly assess the presence of osmotic stress + cell-specific (some metabolites predominantly located in neurons like NAA, Glu or in astrocytes like Gln, mIns) + quantitative, translational + non-invasive and allows *in-vivo* and longitudinal studies in the same animal + provides insights into the chronology of events involved in HE	- no information on the type of edema - low sensitivity of the MRS signal originating from metabolites
Diffusion-weighted magnetic resonance spectroscopy (DW-MRS)	+ probes tissue microstructure at intra-cellular level as metabolites are mainly intra-cellular: changes in intra-cellular metabolites diffusivity, in soma and processes radius, in processes lengths and ramifications + potential information on the type of edema, provided that the results are validated against other *ex-vivo* techniques as done in stroke with DW-MRI + quantitative, translational + non-invasive and allows *in-vivo* and longitudinal studies in the same animal + cell-specific (some metabolites predominantly located in neurons like NAA, Glu or in astrocytes like Gln, mIns) + provides insights into the chronology of events involved in HE	- indirect measurement: requires modelling of metabolites diffusion properties - low sensitivity of the MRS signal originating from metabolites - multiple interpretations possible

**Table 2:** Summary of some published work highlighting the presence of edema in hyperammonemia *in-vitro* and *ex-vivo* HE models

Subject	Type of measurements	Method	Comments	Ref
Rat - hyperammonemia - NH_4_Cl (5 and 10 mM)	*In-vitro*: primary cultures of astrocytes from neonatal cerebral cortex	Light and electron microscopy	Initial response of astrocytes - of hypertrophy (compensatory) followed by degenerative changes Light microscopy - increased cytoplasmic basophilia, cytoplasmic granularity, vacuolization, formation of dense bodies, and eventual cellular disintegration - Alzheimer type-II-like cells were found in areas adjacent to necrosis Electron microscopy - astrocyte treated for 1 d with 5 mM ammonia shows prominent filamentous dense bodies, loss of glial filaments and occasional vacuoles	Norenberg (1987)
Guinea pig (weight 300–350 g) and rat (weight 220–230 g) - hyperammonemia - NH_4_Cl (5 and 10 mM)	*Ex-vivo*: guinea pig and rat cerebellar slices (0.4 mm)	final wet weight vs. initial wet weight of the slices - measure of the increase of water uptake	- guinea pig: brain slices swelling too small to be significant - NH_4_Cl (5 mM and 10 mM) - rat: significant swelling of the brain slices at NH_4_Cl 10 mM - the outward flow of K^+^ was greater than the net inward flow of${NH}_{4}^{+}$- could not be explained as simply due to an exchange - increased the tissue content of Na^+^ and Cl^−^ - presumably enhanced water uptake occurs in the glia as the neurons undergo little swelling	Benjamin et al. (1978)
Rat - hyperammonemia - NH_4_Cl (2, 5, and 10 mM)	*In-vitro*: primary cultures of astrocytes from neonatal cerebral cortex	Methyl-D-glucose, 3-O-[Methyl-^14^ C] and [^3^ H]-3-O-Methylglucose method -${\varvec{\beta }}^{-}$radiation	− 5 mM NH_4_Cl − 12% ↑ in astrocytes volume after 4 days of treatment (p < 0.02). − 10 mM NH_4_Cl - one day of treatment: 11 + 1.4% ↑ (p < 0.001) while treatment for 4 days resulted in a 29 ± 3.0% ↑ (p < 0.0001) in astrocytic water space - after 3 days ammonia-induced swelling was still reversible in normal culture media for 1 day - ammonia ↑ lead to astrocyte swelling and may contribute to the brain edema in fulminant liver failure - of note: cells maintained in fetal calf serum did show some swelling upon treatment with ammonia, the effect was smaller and not as consistent as that seen in horse serum-treated cells − 5 mM NH_4_Cl co-treatment with 0.1 mM aspartate (3 days) - suppressed the ammonia-induced swelling of astrocytes ~ 68% − 1 mM ornithine had no significant effect on the ammonia-induced swelling of astrocytes	Norenberg et al. (1991), Murthy and Norenberg (2002)
Rat - hyperammonemia - NH_4_Cl (5 mM) + antioxidants (SOD, CAT, vit. E) and MAPKs inhibitors SB239063 (an inhibitor of p38-MAPKs), and SP600125 (an inhibitor of c-Jun N-terminal kinase, JNK)	*In-vitro*: primary cultures of astrocytes from neonatal cerebral cortex	Western blots Immunofluorescence of NFκB [^3^ H]-3-O-Methylglucose method -${\varvec{\beta }}^{-}$radiation Fluorescence spectroscopy	- increased astrocyte swelling by 40.2% (p < 0.05, compared to control) - aminoguanidine (500 µM) partially prevented astrocyte swelling, while 1 mM completely blocked it - activation of NFκB after 12 h exposure to ammonia (5 mM) - inhibitors of NFκB, BAY 11-7082 (5 µM) and SN-50 (3 µM) (76.3 and 66.8%, respectively, as compared to control, p < 0.05) diminished NFκB activation by ammonia - SOD (25 units/ml) and vit. E (100 µM) diminished ammonia induced NFκB activation (69.7, 59.8 and 55.0% respectively, p < 0.05) - ammonia-induced astrocyte swelling was inhibited by the NFκB inhibitors BAY 11-7082 and SN-50 - MAPKs inhibitors attenuate NFκB activation by ammonia - NFκB inhibitors diminish ammonia-induced astrocyte swelling - significant role of oxidative stress and MAPKs in NFκB activation - activation of NFκB was associated with increased iNOS protein expression and NO ↑, and these changes were blocked by BAY 11-7082, an inhibitor of NFκB	Sinke et al. (2008)
Rat - hyperammonemia - NH_4_Cl (5 mM) + cytokines - TNF-α, IL-1b, IL-6 and IFN-g, all 10 ng/ml each, either individually or in combination and NF-kB inhibitor (BAY 11-7082)	*In-vitro*: primary cultures of astrocytes from neonatal cerebral cortex	[^3^ H]-3-O-Methylglucose method -${\varvec{\beta }}^{-}$radiation Western blots	- key role of NF-kB in the mechanism of ammonia-induced astrocyte swelling - swelling was increased by ammonia (43%) and by cytokines (37%) at 24 h - co-treatment with cytokines and ammonia showed no additional swelling − 24 h ammonia pretreatment followed by additional 24 h exposure to cytokines → a marked increase in astrocyte swelling ~ 129% - treatment with ammonia or cytokines alone also activated NF-kB (80–130%) − 24 h ammonia pretreatment followed by additional 24 h exposure to cytokines → a marked activation of NF-kB (428%) - NF-kB inhibitor (BAY 11-7082), completely blocked the astrocyte swelling in cultures pre-treated with ammonia and followed by the addition of cytokines	Rao et al. (2010)
Rat - hyperammonemia - NH_4_Cl or NH_4_CH_3_CO_2_ (2, 5 and 10 mM, 1–10 days)	*In-vitro*: Primary cultures of astrocytes from neonatal cerebral cortex	Light and electron microscopy	Acute ammonia intoxication Light microscopy - increase in basophilia - prominent cytoplasmic processes - increased cytoplasmic granularity and microvacuolization - increase in nucleolar/nuclear ratio - fragmentation of cytoplasmic processes - formation of dense bodies - cellular disintegration Electron microscopy - disaggregation of polyribosomal clusters - loss of glia filaments - RER and SER ↑ - mitochondria number and length↑ - mitochondria swelling with intercristal space widening - increased cytoplasmic granularity and microvacuolization	Gregorios et al. (1985a, b)
Rat - hypoosmotic exposure (205 mosmol/L)	*In-vitro*: cultured rat astrocytes - from the cerebral hemispheres of newborn rats	Western blot	- induces MAP kinase activation - important role of extracellular calcium in the osmo-signaling pathway upstream of the MAP kinases - sustained elevation of the intracellular Ca^2+^ concentration - entry from the extracellular space - hypoosmotic astrocyte swelling activates MAP-kinases in a Ras/Raf-dependent and PI3-kinase-dependent way which is initiated by a swelling-induced Ca^2+^ signal	Schliess et al. (1996), Häussinger et al. (1997)
Rat - hyperammonemia - NH_4_Cl (1, 5 and 10 mM)	*Ex-vivo*: brain slices (cortex)	Dry-weight technique Light microscopy	- initial water content 83.03 ± 0.54% (control) → 86.25 ± 1.16% (5 mM) → 88 ± 0.62% (10 mM) - swelling - water accumulation ↑ with ammonia concentration ↑ - increased intracellular Na^+^ and decreased K^+^ content - decrease in the space of distribution of inulin was seen at the 10 mM concentration, suggesting intracellular water accumulation - spongiform change of the neuropil - enlarged astrocytic nuclei, redistribution of chromatin, and a clear nucleoplasm	Ganz et al. (1989)
Rat / mouse – hypoosmolality, hyperammonemia - NH_4_Cl (5 mM)	*In-vitro*: cultured rat astrocytes - from the cortices of newborn rats *Ex-vivo*: brain slices (cortex) from male mice (adult)	Immunostaining Fluorescence microscopy Western Blot	Acute ammonia intoxication - hypoosmotic (205 mosmol/L) swelling of cultured astrocytes induced a rapid generation of ROS - ammonia, similar to hypoosmolality, induced a rapid astrocyte swelling - ROS production by ammonia is accompanied by a rapid astrocyte swelling, which is reversible upon removal of ammonia - close interrelation between astrocyte swelling and oxidative stress	Reinehr et al. (2007)
Rat / mouse – hyperammonemia - NH_4_Cl (5 mM)	*In-vitro*: cultured rat astrocytes - from the cortices of cerebral hemispheres of newborn rats	IHC - confocal laser-scanning microscopy North-Western and Slot-Blot Analysis - isolated RNA	Acute ammonia intoxication - ammonia and hypoosmotic swelling increased RNA oxidation - ammonia-induced RNA oxidation is reversible in-vivo	Gorg et al. (2008)
Rat / mouse – primary astrocytes / neurons - hyperammonemia - NH_4_Cl (5 mM) Rat - hyperammonemia (weight 280 ± 6 g) - NH_4_CH_3_CO_2_ (4.5 mM)	*In-vitro*: cultured rat astrocytes - from the cortices of cerebral hemispheres of newborn Wistar rats co-cultured with hippocampal neurons from embryonic C57BL/6Jmice *Ex-vivo*: brain slices (cortex)	Fluorescence and electron microscopy	*In-vitro*: - moderately increased concentrations of NH4Cl induce autophagy while a high concentration ~ 5 mM / hyperammonemia inhibits autophagic flux in primary astrocytes − 5mM NH_4_Cl → 5-fold increase in the number of autophagosomes/autolysosomes compared to the water-treated control - inhibition of autophagy is largely mediated by changes in intracellular and intralysosomal pH - ammonia-induced inhibition of autophagy occurs in a ROS-dependent manner - neurons show impairment of autophagy under hyperammonemia *Ex-vivo*: -astrocytes and neurons autophagy is impaired by hyperammonemia - varying degree in certain brain regions	Lu et al. (2019)
Mouse – hyperammonemia - NH_4_CH_3_CO_2_ (1–10 mM)	*In-vitro*: post-natal organotypic slice cultures – forebrain	Optical coherence tomography (OCT) - of cultured slices Confocal microscopy Immunoblotting	- ammonia for 3 days → evidence of tissue edema that is linked with astrocytic swelling - no significant changes in slice area were detected over 3 days at ammonia concentrations up to 10 mM - NH_4_CH_3_CO_2_ 10 mM resulted in macroscopic tissue swelling, with slice thickness increasing ~ 30%, treated: 441 ± 40 μm vs. control: 345 ± 33 μm - volume of GFAP-immunoreactivity was used as a marker of astrocyte cytoplasmic volume - the fractional volume of cortex occupied by GFAP- immunoreactive voxels progressively and significantly increased at concentrations of 1–10 mM of NH_4_CH_3_CO_2_ - NH_4_CH_3_CO_2_ 10 mM resulted in 8% increase in volume of GFAP-immunoreactivity - no change in astrocytes number - neurons somatic diameter of control ranged from 10–15 μm vs. 20 μm after treatment with NH_4_CH_3_CO_2_ 10 mM associated with large swollen nuclei	Back et al. (2011)
C6 Glioma Cell line (AmericanType Culture Collection (Rockville, MD)) -hyperammonemia - NH4Cl (5 mM) + resveratrol (100 µM)	*In-vitro*: astroglia cells	Optical microscopy	- ammonia induced astrocyte swelling / cell body retraction, and resveratrol prevented this effect - ammonia induced increased expression of cytokines, the TNF-α, IL-6 and IL-1β, which was partially reversed by resveratrol (TNF-a levels from 148 ± 13% to 109 ± 13%, IL-1β levels from 140 ± 9% to 110 ± 13%, and IL-6 from 135 ± 13% to 115 ± 16%, values expressed as percentage of control)	Bobermin et al. (2012)
Brain cell 3D culture hyperammonemia - NH4Cl (5 mM) + creatine (1 mM)	*In-vitro*: mixed-cell aggregates – from mechanically dissociated telencephalon of 16-day rat embryos	Optical microscopy	- ammonia induced astrocyte swelling, and creatine prevents this effect - NH_4_Cl alters AGAT, GAMT and SLC6A8 expression in glial cells but not in neurons - increased GAMT but repressed SLC6A8 mRNA expression in oligodendrocytes - creatine co-treatment under NH_4_Cl exposure represses AGAT and SLC6A8 in swollen astrocytes - decrease of endogenous synthesis of creatine under NH_4_Cl exposure - ammonia exposure impaired axonal growth in developing mixed cell aggregates - creatine prevented ammonia-induced axonal growth impairment in developing mixed cell aggregates - creatine did not prevent the ammonia-induced axonal growth impairment in developing neuron-enriched aggregates - neuronal fiber growth impairment under ammonium exposure, together with a decrease in medium size neurofilaments (NF-M) - in mature aggregates ammonia exposure does not alter axonal morphology → difference in vulnerability of the developing and adult brain - no significant effect was found for oligodendrocytes	Braissant et al. (2002)

In rodent studies, the most frequently used methods for brain water measurements include the dry/wet weight technique (Marmarou et al. 1978) and the specific gravity method (Hayazaki et al. 1995). While no specific skills or state-of-the-art equipment are required for these methods, just as with the current imaging techniques, the extra- and intracellular water content cannot be distinguished (Bemeur et al. 2016; Cudalbu and Taylor-Robinson 2019). Electron microscopy (EM) offers extracellular space images with nanoscale resolution (Kasthuri et al. 2015). This technique offered the first evidence of a potentially cytotoxic edema, characterized by Alzheimer’s Type II astrocytes, in animals (Norenberg and Lapham 1974) and patients with chronic liver disease (CLD) and HE (Agarwal and Mais 2019). However, the major disadvantage of EM is the need for tissue fixation, making it incompatible with live tissue and real time recordings. Classical chemical fixation dehydrates the sample and effectively disturbs the water distribution in the tissue, leading to cell swelling and shrinkage of the extracellular space (Korogod et al. 2015). While cryofixation immobilises molecules in their hydrated state (reducing structural fixation artifacts), only the outermost few microns of the tissue sample are reportedly artifact-free (Studer et al. 2008), making EM suboptimal for studying brain edema.

**Fig. 2:**
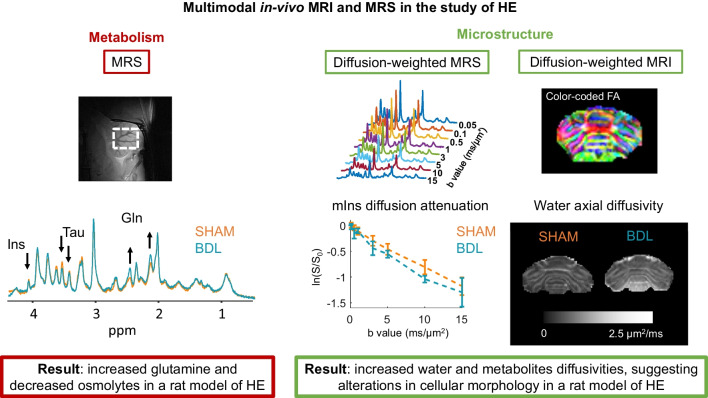
*In-vivo *MRS, DW-MRS and DW-MRI in the study of HE. Left panel – brain metabolism: *In-vivo* ^1^H MRS** -** Representative neurometabolic profiles in one BDL and one Sham rat acquired at 14.1T in the cerebellum. Arrows show significant changes in the BDL group (increase in Gln, decrease in osmolytes). Right panel - microstructure: DW-MRS** -** Representative spectra at different b-values and intra-cellular diffusion attenuation of mIns in the BDL and Sham groups, showing faster mIns diffusivity in BDL rats (as measured from, for e.g., the randomly oriented sticks model (Callaghan et al. 1979)). DW-MRI** -** Color coded fractional anisotropy map of an axial slice through the body of cerebellum. Water diffusivity is also increased in BDL rats.

Brain water mapping using MRI (Shah et al. 2008; Winterdahl et al. 2019) has been shown to be the most precise non-invasive method that can be applied to patients with HE for absolute quantification of cerebral hydration status in all brain regions (e.g., 1% change in water content have been detected in HE patients) (Oeltzschner et al. 2016; Shah et al. 2008). However, it lacks specificity regarding the etiology of water accumulation (Bemeur et al. 2016). Some other *in-vivo* and non-invasive methods like diffusion-weighted MRI (DW-MRI), a powerful tool to probe brain microstructure, allow the non-invasive assessment of the microstructural changes in the brain during HE (e.g., extra- and intra-cellular water diffusivity and fraction, soma and processes radius, orientation dispersion of fiber bundles) without a firm conclusion on the absolute water content or on the cell-specificity of these changes (Fig. 2). Water diffusion is hindered or restricted by the brain cellular environment, resulting in a deviation of the MR signal diffusion attenuation from the one expected for free diffusion. With appropriate modelling, the study of these diffusion properties can inform on brain tissue morphology. In particular, the technique is known to be sensitive to subtypes of brain edema especially in stroke, where DW-MRI findings were validated against complementary *ex-vivo* methods (Benveniste et al. 1992; Moseley et al. 1990): cytotoxic edema (associated with a decreased apparent diffusion coefficient (ADC)) versus vasogenic edema (associated with increased ADC) (Ebisu et al. 1993; Ito et al. 1996). This interpretation of DW-MRI metrics requires validations by specific *ex-vivo*/histological measures when using new animal models. Moreover, deciphering the underlying mechanism resulting in a decreased ADC remains challenging: it could be due to greater contribution of the slowly-diffusing intracellular space (Benveniste et al. 1992), increased tortuosity of the extracellular space (Latour et al. 1994), neurite beading (Budde and Frank 2010), increased cytoplasmic viscosity (Goodman et al. 2008) or increased fraction of membrane-bound water (Jelescu et al. 2014). These multiple causes contribute to some limitations of the technique to inform on edema. Recently, several biophysical models have been developed in the attempt to overcome these limitations (Jelescu et al. 2020). Most literature in cirrhosis patients with or without HE reports an increased water ADC or mean diffusivity (MD) (Chavarria et al. 2011, 2013a; Kale et al. 2006; Lodi et al. 2004; Mardini et al. 2011). These findings are supported by a recent study in bile-duct ligated (BDL) rats where an increased water MD was also observed at week 6 post-surgery, with respect to Sham-operated rats (Mosso et al. 2022a). *In-vivo* proton magnetic resonance spectroscopy studies (^1^H MRS) were among the first *in-vivo* studied used to shed light on the presence of brain Gln increase leading to the presence of osmotic stress (decreased brain myo-inositol (mIns), total choline (tCho), taurine (Tau), creatine (Cr) in type C HE (Braissant et al. 2019; Rackayova et al. 2020; Kreis et al. 1992) which was linked to the development of low-grade brain edema (Haussinger 2006; Haussinger et al. 1994) (Fig. 2). Diffusion-weighted MR spectroscopy (DW-MRS), which studies the diffusion properties of metabolites that are generally observed in ^1^H MRS, offers a greater cell-specificity than DW-MRI (Fig. 2). Some metabolites are known to be mostly astrocytic (mIns, Gln) while others mostly neuronal (N-acetylaspartate (NAA), glutamate (Glu)). DW-MRS could thus allow to study edema at the cellular level. The first DW-MRS study on type C HE showed an increased ADC of some glial (mIns) and neuronal metabolites (Glu) as well as of some osmolytes (mIns, Tau) in adult (Mosso et al. 2021) andyoung (BDL surgery at 21 days after birth) BDL rats at week 6 post-surgery (Mosso et al. 2022a).

In summary, to date there is no “absolute” method to assess brain edema and directly quantify the absolute water content at the cellular level. Overall, current techniques are either only available post-mortem or lack the required resolution and specificity to directly answer the pending question of whether edema in type C HE is of cytotoxic and/or vasogenic origin. In order to address this, both the extracellular space and cell volume/morphology need to be assessed, while methods with increased specificity, resolution and precision are required especially in type C HE where water content changes are small. To overcome these limitations, one solution could be the usage of a multimodal approach by performing *in-vivo* combined with *ex-vivo* experiments for brain water content measurements and for structural cellular changes. This approach would allow monitoring of the progression of the disease longitudinally, providing additional information on the temporal resolution of the onset of brain edema. The assessment of brain regional differences in HE is also crucial since some studies pointed towards a brain regional difference (Cudalbu and Taylor-Robinson 2019), which might explain some of the neurological dysfunction in HE, especially in type C HE.

## What we learned from cell studies: ammonia toxicity *in-vitro*

Although the cell disease models are intrinsically limited, *i.e.*, they lack the complex structure, cell type heterogeneity / in-situ environment (lack of vascular delivery system and BBB) and physical history of diseased tissues, astrocytic cultures are extensively utilized to investigate the primary effects of ammonia (hyperammonemia) on brain (Gorg et al. 2008; Gregorios et al. 1985a, b; Häussinger et al. 1997; Lu et al. 2019; Murthy and Norenberg 2002; Norenberg 1987; Norenberg et al. 1991; Rao et al. 2010; Schliess et al. 1996). It is known that astrocytes protect neurons from ammonia toxicity and the importance of structural complexity in *in-vitro* studies as a model of HE was demonstrated in studies on astrocytes-neurons co-culture (Rao et al. 2005).

In *in-vitro* studies of ammonia treated astrocytes, intracellular water content varies between reports and ammonia concentrations. Treatment with 5 mM ammonia increased cell volume by 12–43% (Murthy and Norenberg 2002; Norenberg et al. 1991; Rao et al. 2010; Sinke et al. 2008). Difference in cellular edema was also related to the applied serum. Astrocytic cultures maintained in fetal calf serum did show some swelling upon pathological treatment with ammonia, but the effect was smaller and not as consistent as that seen in horse serum-treated cells (Norenberg et al. 1991). Studies have shown that co-treatment with 0.1 mM aspartate suppressed the ammonia-induced swelling of astrocytes by 68%, while 1 mM ornithine had no significant effect on cell morphology (Murthy and Norenberg 2002).

In *ex-vivo* of brain slices exposed to ammonia, water content increased in a concentration dependent manner, with 5 mM ammonia increasing the water load by 3% and 10 mM ammonia by 6% (Ganz et al. 1989). Swelling effect differed also between species (Benjamin et al. 1978). Guinea pig brain slices exposed to both 5- and 10-mM ammonia did not show significant swelling, while rat brain slices showed significant swelling only when treated with 10 mM ammonia (Benjamin et al. 1978).

Other studies of *ex-vivo* brain slices exposed to 10 mM ammonia have shown even more pronounced swelling, with slice thickness increasing by ~ 30% (441 ± 40 μm vs. control: 345 ± 33 μm) (Back et al. 2011). Among the 30% increase, only 8% was associated with astrocytic volume changes. Tissue stained with glial fibrillary acidic protein (GFAP) antibody as an astrocyte cytoplasmic fractional volume marker, have shown an increase of immunoreactive voxels (~ 8%) in z-axis (depth), with no change in GFAP content detected (Back et al. 2011).

Increased water uptake in *ex-vivo* brain slices exposed to toxic levels of ammonia (5–10 mM) was accompanied by an increased tissue content of Na^+^ and Cl^−^ and loss of K^+^ (Benjamin et al. 1978), which was suggested to be related to sodium pump impairment as a result of energy failure (Gregorios et al. 1985a).

*In-vitro* ammonia induced cell swelling and elevation of intracellular osmolarity may be related to the accumulation of Gln and Ca^2+^ content reduction (Norenberg et al. 1991). Treatment with extracellular adenosine triphosphate (ATP) showed increased Ca^2+^ influx and accumulation and resulted in reduction of ammonia induced astrocyte swelling (Norenberg et al. 1991). Cell swelling has been also linked to a variety of mechanisms, among which are defects in: ion pumps/channels (Na^+^/K^+^), cotransporters (Na^+^/K^+^/Cl^−^), release of osmotically active amino acids and the buildup of osmotically active compounds (Norenberg et al. 1991). Increased Gln production in hyperammonemia deprives the cytosolic glutamate pool, compromising the activity of the malate–aspartate shuttle (MAS), which transports reducing equivalents from the cytosol into the mitochondria (Murthy and Norenberg 2002). Furthermore, cytosolic Glu depletion in the presence of ammonia inhibits pyruvate oxidation and its further entry into the citric acid cycle, resulting in pyruvate to lactate conversion for nicotinamide adenine dinucleotide (NAD) from NADH regeneration and glycolysis continuation (Murthy and Hertz 1988; Murthy and Norenberg 2002).

*In-vitro* findings indicated activation of mitogen-activated protein kinase (MAPKs) in a Rat Sarcoma Virus (Ras) / Rapidly Accelerated Fibrosarcoma (Raf)- and phosphatidylinositol 3-kinases (PI3-kinase)-dependent manner, triggered by swelling-induced Ca^2+^ signal in astrocytes exposed to a hypoosmotic environment (Häussinger et al. 1997). In addition, hypoosmotic shock leads to a rapid release of osmolytes (mIns and Tau) most likely via the opening of unspecific channels in the plasma membrane (Häussinger et al. 1997).

The synchronous treatment of astrocytes with hazardous doses of ammonia and cytokines (tumor necrosis factor-α (TNF-α), interleukin 1β (IL-1β), interleukin 6 (IL-6) and interferon-γ (IFN-γ)) had no additive or synergistic impact on swelling. On the other hand, astrocytic swelling increased after a 24-hour pretreatment with ammonia followed by a 24-hour exposure to cytokines (Rao et al. 2010). In addition, exposure to both, ammonia and cytokines, separately or simultaneously, induce the nuclear factor-κB (NF-κB) activation (Rao et al. 2010), which thereafter will influence cell survival in a complex way (neuroprotective or proinflammatory), depending on the pathological stage (Back et al. 2011). NF-κB activation suppression by NF-κB and MAPKs inhibitors, and antioxidants (superoxide dismutase (SOD), vit. E) prevented astrocytic swelling in cultures pre-treated with ammonia and followed by cytokines (Rao et al. 2010; Sinke et al. 2008). These studies indicate a pivotal role of NF-κB in the potentiation of cellular edema. NF-κB activation blockage was also associated with a reduction in the ammonia-induced increase in oxidative/nitrosative stress, inducible nitric oxide synthase (iNOS) protein expression and nitric oxide (NO) generation, plausibly one of the main factor of the ammonia-triggered astrocytic swelling mechanism (Rao et al. 2010; Sinke et al. 2008).

A recent *in-vitro* and *ex-vivo* study has indicated that even low concentrations of ammonia (1–5 µM) can induce reactive oxygen species (ROS) thus leading to neuronal cell death (Angelova et al. 2022).

*In-vivo* longitudinal antioxidant system impairment indicated the presence of OS (Braissant et al. 2019). The observed ascorbate (Asc) decrease (Braissant et al. 2019) can be related to the increased CNS and liver ROS levels (Pierzchala et al. 2022; Simicic et al. 2022), compromised Asc synthesis due to diseased liver (Linster and Van Schaftingen 2007), and decreased exogenous availability related to nutrient deficiency (Ipsen et al. 2014). *Ex-vivo* longitudinal CNS OS detection demonstrated an increased superoxide anion (${\text{O}}_{2}^{.-}$) production in BDL rats, which was due to enhanced formation of ROS rather than a decrease of antioxidants activity (elevated levels of SOD1/2 and GPX-1 were observed) (Pierzchala et al. 2022).

As a more complex system to simple cell cultures, regular brain cell 3D cultures (*i.e.*, mixed-cell cultures) prepared from the telencephalon of rat embryos have also been challenged with ammonia (5 mM) (Braissant et al. 2008; Braissant et al. 2002). In 3D brain cell culture, ammonia caused a decrease of endogenous synthesis of creatine and induced astrocyte swelling. Creatine co-treatment prevented this effect (Braissant et al. 2002).

The majority of the studies published till now focused on astrocytes (Table 2) and showed an ammonia concentration dependent (5–10 mM) swelling/edema. The organotypic slice model of ammonia-induced (10 mM) brain swelling additionally demonstrated an increase of neuronal soma diameter associated with large swollen nuclei (Back et al. 2011). It is important to highlight that the majority of ammonia levels used in these studies might have been too high and thus not physiologically relevant for both type A and C HE. Therefore, it remains to conclude if such high ammonia concentrations are required to induce swelling/edema or if the detection techniques used to date were not sensitive enough to detect edema/swelling at lower ammonia concentrations. Measuring brain tissue ammonia levels is difficult due to the multiple preanalytical steps (i.e., extraction of the brain, postmortem metabolic changes, the detection technique) with cerebrospinal fluid (CSF) and microdialysates measurements of ammonia being performed more often (DeMorrow et al. 2021). We encourage the development/validation of new techniques to measure brain ammonia in type A and C HE and afterwards establish if these levels of ammonia would lead to the same amount of swelling/edema. Moreover, an experimental correlation between brain, blood and CSF ammonia levels in the same animal model is highly warranted for the HE community and worth considering in the future. Additional works should also focus on characterizing other cell types and not only astrocytes.

## What we learned from animal models of type A HE

Ammonia neurotoxic outcomes are based on glial and neuronal cell dysfunction (Braissant et al. 2019; Rangroo Thrane et al. 2013). *In-vivo* type A HE manifests with a progressive increase in the water content of cortical gray matter, astrocyte morphology alterations/swelling of an intracellular compartment (Blei et al. 1994; Master et al. 1999; Norenberg 1977, 1987; Norenberg and Lapham 1974; Rangroo Thrane et al. 2013; Swain et al. 1991, 1992; Traber et al. 1986, 1987, 1989; Zamora et al. 1973) as well as a strong link between astrocyte swelling and OS (Reinehr et al. 2007).

Overall, based on *in-vitro* studies, neurotoxicity may rise not just from the consequences of tissue/astrocyte swelling and elevated ICP, but also from acute ammonia toxicity to neurons, in particular when extremely high ammonia levels are persisted for a longer period (Back et al. 2011). Given the fact that GS in the brain is located mainly in astrocytes, its role is to protect neurons from excitotoxicity caused by excess of ammonia by converting it into Gln in the presence of Glu and ATP (Suarez et al. 2002; Traber et al. 1989). However, the potential to improve ammonia detoxification by GS is limited (Butterworth et al. 1988). Therefore, the protection of neurons by astrocytes may be compromised, and accumulation of water and cytotoxic compounds, among which are ROS and reactive nitrogen species (RNS) (Pierzchala et al. 2022), would cause deleterious effects on neurological function.

Similar to the results obtained from brain slices exposed to the toxic levels of ammonia (Benjamin et al. 1978), *in-vivo* studies confirmed that ammonia has an impact on astrocyte potassium buffering, leading to a rise in extracellular ${\left[{\text{K}}^{+}\right]}_{\text{o}}$ (~ 2 mM) with immediate and acute neurological deterioration (seizures in awake animals) (Rangroo Thrane et al. 2013). Excess of both ${\left[{\text{N}\text{H}}_{4}^{+}\right]}_{\text{o}}$ and ${\left[{\text{K}}^{+}\right]}_{\text{o}}$ promotes further overactivation of the Na^+^-K^+^-2Cl^−^ cotransporter isoform 1 (NKCC1) in neurons and selectively impairs cortical inhibitory neurotransmission, while NKCC1 inhibition reduces the neurological symptoms of acute ammonia intoxication (Rangroo Thrane et al. 2013). Furthermore, inhibition of GS worsened the neurological outcome by increasing the ${\left[{\text{N}\text{H}}_{4}^{+}\right]}_{\text{o}}$ and ${\left[{\text{K}}^{+}\right]}_{\text{o}}$ load on neurons (Rangroo Thrane et al. 2013).

EM studies on the brains of type A HE animal models disclosed morphological changes of astrocytes. In the early phase represented by cellular hypertrophy, an increased number of cytoplasmic organelles including mitochondria, rough and smooth endoplasmic reticulum was observed (Norenberg 1977). This early reaction may occur to preserve cellular homeostasis and ammonia detoxification. During the late/coma phase, astrocyte cytoplasmic vacuolization and mitochondrial contraction (Norenberg 1977; Schliess et al. 2009) is a direct sign of inclined deterioration of their protective role, changes in synaptic transmission, and cell death (Master et al. 1999; Shubin et al. 2016).

Methionine-sulfoximine (MSO), an irreversible GS inhibitor, has been shown to avoid the negative effects of hyperammonemia on brain glucose consumption, improve the degree of cerebral edema and the rise in CBF, and prevent intracellular Gln buildup in a dose-dependent manner in the portocaval anastomosis (PCA) model with intravenous infusion of ammonia (Master et al. 1999). The decrease of intracellular Gln was accompanied by an increase in brain Glu levels, as well as plasma and CSF ammonia concentrations (Master et al. 1999).

Brain edema occurs rapidly in type A HE animal models. According to published data (Table 3), brain edema, predominantly attributable to astrocyte swelling, is the major neuropathological hallmark of ALF and plays a critical role in the development of high ICP (Traber et al. 1986) which is related to pathophysiological ammonia levels. Furthermore, there is a significant correlation between Gln levels in the blood, brain, and CSF and the level of ICP in ALF. Additionally, acute Aquaporin 4 (Aqp4) protein rise in the brain is unfavorable, indicating a higher risk of brain edema.

**Table 3:** Summary of some published work highlighting the presence of edema in hyperammonemia animal models – type A HE

Subject	Type of measurements	Method	Comments	Ref
Rat - fulminant hepatic failure - portacaval anastomosis followed by ammoniate resin feedings (coma in 4–10 days)	*Ex-vivo*: cerebral cortex	Light and electron microscopy	Astrocytes: - cellular hypertrophy (increased mitochondria and rough endoplasmic reticulum) - late phase/coma - degenerative changes, cytoplasmic enlargement - proliferation of mitochondria and endoplasmic reticulum, - accumulation of cytoplasmic glycogen, - gliopathy - appearance of the Alzheimer type II change - hydropic alterations as well as degeneration and contraction of mitochondria - swollen astrocyte endfoot - elevation in glutamic dehydrogenase activity in astrocytes	Norenberg (1987), Norenberg and Lapham (1974), Norenberg (1977)
Rat - fulminant hepatic failure (weight 325–425 g) - portacaval anastomosis + NH_4_CH_3_CO_2_ (55 mmol/L/kg/min) − 3-3.5 h of infusion) and intraperitoneal injection of MSO (150 mg/kg)	*Ex-vivo*: cerebral cortex	Gravimetry - cortex punch – ~10 mg	Water content: − 79.97 ± 0.04% in the PCA control group vs. 81.11 ± 0.13% in the NH_3_ group - MSO group 80.03 ± 0.05% and the MSO + NH_3_ 80.48 ± 0.11% - the ammonia group exhibited brain edema - ICP rose significantly in the ammonia-infused group	Master et al. (1999)
Rat - fulminant hepatic failure (weight 300–350 g) - portacaval anastomosis - hepatic artery ligation - portacaval anastomosis and hepatic artery ligation	*Ex-vivo*: cerebral cortex	Gravimetry − 2-mm slices, and 1-mm punch biopsy specimens – cortex UV-Vis – ammonia - glutamate dehydrogenase reaction	- cortical gray matter water ↑ from 80.26% ± 0.22–82.46% ± 0.06% (last stage of devascularization). Tissue preservation without microwaving or snap freezing. - cerebral cortex: brain ammonia ↑ to 5.4 mmol/L. - glutamine ↑ sixfold to 24 mmol/L and remained at this level throughout all stages of encephalopathy	Swain et al. (1992)
Rat - fulminant hepatic failure (weight 300–400 g) - portacaval anastomosis - portacaval anastomosis and hepatic artery ligation	*Ex-vivo*: hemispheres	Gravimetry and the dry-weight technique in whole cerebral hemispheres – 2 mm diameter needle cortex punch Electron microscopy - cortex, cerebellum, and basal ganglia	- marked reduction in body temperature and blood glucose - temperature stabilization and glucose supplementation Water content: - *cortical gray matter* - control animals had a water content of 80.06% ± 0.22%, the IFHF-A group 80.42% ± 0.26%, and the IFHF-B group 81.29% ± 0.38% (p < 0.001). - *brainstem* - control animals had a water content of 77.62% ± 0.79%, the IFHF-A group 77.55% ± 0.48%, and the IFHF-B group 78.35% ± 0.73% (ns). - *cerebellum* - control animals had a water content of 78.87% ± 1.23%, the IFHF-A group 77.75% ± 1.61%, and the IFHF-B group 80.17% ± 0.54% (p < 0.05). Astrocytes: - changes in the cortical gray matter were noted in both groups, coupled with the presence of vesicles containing horseradish peroxidase in the endothelial capillary cell. - astrocytes of the cortical gray matter - variable degrees of cytoplasmic vacuolation and swelling of pericapillary endfoot - brain edema may be due to both a cytotoxic mechanism and changes in the permeability of the blood-brain barrier.	Traber et al. (1989)
Rat - fulminant hepatic failure (weight 200–300 g) - portacaval anastomosis - experiments @ 1–16 weeks post-op ammonia*: ~230 µM	*Ex-vivo*: brain	Light and electron microscopy	- *dentate nucleus* and the caudate nucleus changes were always more marked and usually developed earlier than in other areas - *cerebellar cortex* always showed greater degrees of swelling than elsewhere - initial phase of swelling in all areas, followed by reactive changes in organelles that came to dominate the cytological picture - astrocytes showed considerable swelling of their bodies, processes, and end-feet - mitochondria, endoplasmic reticulum, and ribosomes are increased - dense bodies and lipid droplets and glycogen clumps present	Zamora et al. (1973)
Rat - fulminant hepatic failure (weight 350–450 g) - portacaval anastomosis + NH_4_CH_3_CO_2_ (55 mmol/kg/min, 199 ± 14 min of infusion) – intravenous infusion	*Ex-vivo*: brain	Gravimetry - cortex punch – ~10 mg HPLC and fluorescence detection - glutamine and glutamate content in cortex	Water content ofcCortical gray matter: - significant increase in brain water in both normal and PCA rats after NH_4_CH_3_CO_2_ infusion (before ~ 79.7% (Sham, PCA) and after ~ 80.25% - Sham and ~ 80.94% - PCA (p < 0.001 vs. control)) - significant rise in ICP in PCA rats after NH_4_CH_3_CO_2_ infusion - no change in serum osmolarity Tissue preservation without microwaving or snap freezing. - normal rats NH_4_CH_3_CO_2_ infusion → threefold rise in glutamine, with glutamate levels ↓ - PCA rats - control infusion (CH_3_CO_2_Na) → glutamine ↑ twofold - PCA + NH_4_CH_3_CO_2_ → glutamine ↑~ fivefold - glutamate levels were significantly lower in both groups (Sham, PCA) after NH_4_CH_3_CO_2_ infusion - PCA CSF ammonia: control infusion (CH_3_CO_2_Na) ~ 308 µM, ammonium infusion (NH_4_CH_3_CO_2_) ~ 582 µM	Blei et al. (1994)
Rabbit - fulminant hepatic failure (weight 2–2.6 kg) - galactosamine-induced (4.25 mmol/kg)	*Ex-vivo*: brain	Gravimetry – 2 mm needle diameter punch (~ 10 mg) – cortical gray matter, subcortical white matter, hippocampus, mesencephalic white matter, pontine white matter, cerebellum Dry Weight Method – cortical gray matter (~ 200 mg) Electron microscopy: gray matter of cortex and cerebellum, subcortical white matter and basal ganglia	Water Content: Gravimetry - cortex: control 80.96% ±0.49% vs. mild encephalopathy 81.96% ± 0.47% (p < 0.01) vs. severe encephalopathy 82.95% ± 1.49% (p < 0.01) - hippocampus: control 82.63% ±0.46% vs. mild encephalopathy 81.96% ± 0.47% vs. severe encephalopathy 84.23% ± 0.46% (p < 0.01) - cerebellum, subcortical, mesencephalic, and pontine white matter in encephalopathic animals did not accumulate water Dry Weight Method - cortex: control 79.69% ± 1.7% vs. severe encephalopathy 82.86% ± 0.62% (p = 0.05) Electron microscopy - ultrastructural abnormalities just in gray matter astrocytes, with the most prominent changes in the cerebral cortex - swelling of the pericapillary foot processes of astrocytes - vacuolation in the perinuclear area of the astrocyte cytoplasm - swelling of astrocyte processes surrounding axons and dendrites - swelling of mitochondria not only in astrocytes but also in neurons - axons - mild floccular changes in axoplasm	Traber et al. (1986, 1987)
Rat - fulminant hepatic failure (weight 350–400 g, 6 days) - portacaval anastomosis	*Ex-vivo*: brain	Electron microscopy Electrophysiology	Electron microscopy - astrocytic feet swelling - pericapillary astrocytic area was 7 ± 4 µm^2^ in Sham-operated control animals and 27 ± 26 µm^2^ in PCA animals Electrophysiology - PCA resting membrane potential of 72 ± 5 mV (range: 65–83 mV) and intracellular pH of 7.11 ± 0.11 (range: 6.85–7.34) vs. Sham membrane potential of 81 ± 6 mV (range: 70–93 mV) and a pH of 7.00 ± 0.10 (range: 6.83–7.12) - PCA astrocytes are more depolarized (P < 0.001) and more alkaline (P < 0.009)	Swain et al. (1991)
Rat - fulminant hepatic failure (weight 200–250 g, 6 days) - Thioacetamide (TAA) -induced hepatic encephalopathy:(300 mg/kg i.p) was given to animals daily for 3 days - L-histidine (100 mg/kg): (i.p.) daily 2 h before each TAA injection	*Ex-vivo*: brain	Dry Weight Method – cortical gray matter (~ 10 mg) Brain Ammonia, Glutamine and Glutamine Synthetase	Dry Weight Method - rats treated with TAA showed a 2.9% (P < 0.01) increase in brain water content vs. saline-treated controls (controls and were control: 78.7 ± 0.5% vs. TAA-treated rats: 81.05 ± 0.9%) - L-histidine (25, 50 and 100 mg/kg) completely inhibited the brain edema - L-histidine treatment did not protect against TAA-induced liver injury - threefold increase in brain ammonia levels vs. control − 2.5-fold increase in brain glutamine levels vs. control − 30% (P < 0.05) reduction in GS activity in rats with ALF (despite increased glutamine levels)	Rama Rao et al. (2010)
Rat - fulminant hepatic failure (weight 270–300 g) - galactosamine-induced (0.4 M (85 mg/mL))	*Ex-vivo*: brain	Light and electron microscopy Dry Weight Method – whole brain BBB permeability	Dry Weight Method − 4% brain swelling in 24 h Microscopy - evidence of cerebral and cerebellar edema (perivascular region) - swelling and distortion of astrocytes including subcellular organelles (I.e., mitochondria) - cytotoxic component preceding the vasogenic edema - breakdown of BBB during progressive stages of liver failure	Dixit and Chang (1990)
Male C57 BL/6 mice - fulminant hepatic failure (weight 25–26 g) - galactosamine-induced (800 mg/kg) and LPS Escherichia coli 0111:B4 (10 µg/kg) NH_4_ inffussion 120 µmol/kg/min (100 µl/hour): 6.5 h - total experimental time	*Ex-vivo*: brain	Gravimetry – cortex: 2-mm slices Western blotting and dot blotting	Water Content: Gravimetry - significantly increased brain water content in the GLN + LPS + NH_4_ group: 80.8 ± 0.29% vs. control: 80.0 ± 0.13% (p < 0.05) Western blotting - Aqp1 expression increase of 1.18 times (GLN + LPS + NH_4_ group vs. control) - Aqp4 expression increase of 1.64 times (GLN + LPS + NH_4_ group vs. control)	Eefsen et al. (2010)
Rat - fulminant hepatic failure (weight 250–300 g, 6 days - portacaval anastomosis + hepatic artery ligation (HAL)	*In-vivo*: brain *Ex-vivo*: cerebral cortex	DTI and ^1^H MRS (7T) - intracellular or extracellular distribution of brain water and metabolites Gravimetry – cortex: 2-mm slices BBB permeability – cortex 100 mg − 25 µCi [U-^14^ C] sucrose – scintillation counting	DTI and ^1^H MRS - PCA rats did not show any change either in T2 or ADC values in all the studied regions - ALF rats did not show any change in T2 but did show a decrease in ADC in all regions - ALF vs. Sham-operated controls: the brain-ADC in ALF was 8% lower after 6 h (P < 0.053), 14% lower at precoma (P < 0.030), and 20% lower at coma stage (P < 0.001) - glutamate in ALF was lower than in Sham rats - choline derivates had decreased compared with Sham - cytotoxic mechanism: ammonia in astrocytes induces an increase of glutamine and lactate that appears to mediate cellular swelling Water Content Gravimetry - percentage of cerebral water content: in Sham rats (81.6% ± 0.3%), all rats with ALF at 6 h (81.8% ± 0.2%), precoma (82.0% ± 0.3%), and coma (82.3% ± 0.4%) - brain water content increase was significant at the coma stage (P < 0.05) BBB permeability - cytotoxic origin of brain edema was supported by the lack of increase in the BBB permeability to ^14^ C-sucrose.	Chavarria et al. (2010)

*Ammonia measured before sacrifice

In addition to type A HE animal models, recent *in-vivo* hyperammonemia studies (two-photon microscopy) have shown no astrocyte swelling or brain edema in the acute phase of ammonia increase. Instead astrocyte shrinkage was observed lasting for 30 min after intraperitoneal (i.p.) ammonia injection (Rangroo Thrane et al. 2013). To induce brain edema and astrocyte swelling (50 mM in-situ), a lethal dose of ammonia (10 mmol/kg i.p.) was required (Rangroo Thrane et al. 2013).

## What we learned from type C HE

### *In-vivo ***– **animal models of type C HE

Several studies have investigated the changes in brain water content in animal models of type C HE, with the BDL model being the widest used, a model endorsed by ISHEN (DeMorrow et al. 2021). The results of these studies are summarized in Table 4. As indicated, the gravimetry technique seems to be the most used. Nonetheless, there are only a few published studies using these techniques, and the results appear to be controversial, with studies reporting changes in BDL rats while others not. Of note, the changes reported are small, e.g., 1–2% increase in water content, similar with what was measured in humans using absolute water measurements with MRI. Complementary methods to evaluate the hydration status of the brain might be useful. MRI and MRS offer the possibility to evaluated *in-vivo* and longitudinally changes in brain structure/microstructure, volumes, osmoregulation, metabolism and can be used complementary to any *ex-vivo* end point measurement as gravimetry or EM. The increase in brain Gln was a consistent finding in the majority of type C HE work published till now, in animal models but also in humans. In addition, a decrease of other brain osmolytes, as an osmotic counteraction of the increase in brain Gln, was also measured (Table 4). These findings were interpreted to reflect a volume regulatory response to compensate for Gln induced osmotic imbalance in astrocytes, as Gln is considered to be mainly located in astrocytes. It has been speculated that there is a brain regional difference in type C HE (Mosso et al. 2022b; Flatt et al. 2021), therefore it would be interesting to establish if brain metabolism/microstructure and other parameters are brain region dependent, together with possible changes in the morphology/structure of astrocytes, neurons, microglia and endothelial cells. These studies would be instrumental in identifying the ammonia fingerprints in the brain.

**Table 4:** Summary of some published work highlighting the presence of edema in animal models of type C HE

Subject	Type of measurements	Method	Comments	Ref
BDL rat model at 6 weeks post-op (weight 250–275 g) Arterial ammonia - BDL* 119.7 ± 15.2 µM CSF ammonia - BDL* 128.4 ± 36.7 µM	*Ex-vivo*: cerebral cortex	Gravimetry − 2-mm^2^ slices - frontal cortex	Water Content: - water content in frontal cortex was significantly increased in BDL rats 79.46 ± 0.28% vs. BDL-Sham 78.35 ± 0.17%, p < 0.05	Bosoi et al. (2012)
BDL rat model (weight ~ 320 g) − 4 weeks post-op - injected with LPS (0.5 mg/kg, intraperitoneally) → 3 h later sacrificed Plasma ammonia*: BDL: 168 ± 14 µM BDL + LPS: 172 ± 37 µM	*Ex-vivo*: brain	Gravimetry − 2-mm^2^ slices - frontal cortex Electron microscopy - frontal cortex	- LPS induced cytotoxic brain swelling - TNF-α, IL-6, and plasma nitrite/nitrate levels significantly increased in LPS-treated animals - Plasma TNF-α (nmol/L): Sham 0.03 ± 0.02 vs. BDL 0.05 ± 0.02 - Plasma IL-6 (nmol/L): Sham 0.053 ± 0.005 vs. BDL 0.094 ± 0.035 - Brain TNF-α (nmol/L): Sham 0.008 ± 0.001 vs. BDL 0.011 ± 0.002 - Brain IL-6 (nmol/L): Sham 1.3 ± 0.24 vs. BDL 2.1 ± 0.2 Water Content: Gravimetry - frontal cortex: significant increase was seen in rats administered with LPS, both Sham and BDL. Sham: 79.8% ± 0.29%, Sham + LPS: 80.9% ± 0.24%, BDL: 79.9% ± 0.27%, BDL + LPS: 80.8% ± 0.18% → no difference between Sham + LPS vs. BDL + LPS Tissue preservation without microwaving or snap freezing Brain ammonia (µmol/g ww) – Sham: 0.27 ± 0.08, Sham + LPS: 1.2 ± 0.17, BDL: 1.0 ± 0.36, BDL + LPS: 2.0 ± 0.66 Electron microscopy BDL - partially collapsed microvessel and minimal water accumulation in the astrocytic feet and perivascular tissue BDL + LPS - massive astrocytic feet and perivascular edema and collapsed microvessel	Wright et al. (2007a)
BDL rat model (weight 220–250 g) – 4 weeks post-op Plasma ammonia*: 194.45 ± 4.89 µM	*Ex-vivo*: mitochondria – 4w post-op, brains were removed - cortex, hippocampus, striatum, and cerebellum - homogenized	UV-Vis and fluorescence spectroscopy	Permeability transition pore opening → swelling Mitochondria from cortex, hippocampus, striatum, and cerebellum of BDL rats: - cerebellum ~ 59% - cortex ~ 55% - hippocampus ~ 37% - no significant swelling in striatum	Dhanda et al. (2018)
BDL rats @ 4, 5, 6 weeks post-BDL Adult SD rats	*In-vivo*: brain MRS and DW-MRI experiments *Ex-vivo*: brain	7T, ^1^H MRS, PRESS, TE = 12 ms, voxel 6.5 × 6.5 × 6.5 mm^3^ DTI, 20 directions and 4 b-values VC, SC, MC, Hip, Tha, HypoT, Str, NC Gravimetry	↑ Gln ↓ Glu, tCho, tCr, NAA and mIns No change in Lac No difference in ADC values between BDL and sham operated rats and neither in water content using gravimetry	Chavarria et al. (2013b)
BDL rats @ 0, 4, 8 weeks post-BDL Adult Wistar rats Plasma ammonia*: 157 ± 100 µM	*In-vivo*: brain	9.4T, ^1^H MRS, SPECIAL, TE = 2.8 ms 4 × 7.5 × 6.5 mm^3^ ^31^P MRS	↑ Gln and plasma NH4 + post-BDL ↓ mIns, tCho @ 8 weeks post-BDL ↓ Glu, Asp @ 8 weeks post-BDL ↑ Sum of all measured main brain organic osmolytes (Gln + mIns + tCho + Cr) @ 8 weeks post-BDL mild alterations in energy metabolism but only at the end stage of the disease	Rackayova et al. (2016)
BDL rats @ 0, 2, 4, 6, 8 weeks post-BDL Adult Wistar rats	*In-vivo*: brain *Ex-vivo*: brain	9.4T, ^1^H MRS, SPECIAL, TE = 2.8 ms 2 × 2.8 × 2 mm^3^ - hippocampus Fluorescence microscopy GFAP	↑ Gln and plasma NH4+ @ 2 weeks post-BDL ↓ Cr and Asc @ 2–4 weeks post-BDL ↓ mIns, Tau, tCho @ 6 weeks post-BDL ↓ Glu @ 8 weeks post-BDL Astrocytes: - time-dependent changes in astrocyte morphology visible already @ 4 weeks post-BDL - astrocytes showed a thickening of main proximal processes, as well as their retraction - time-dependent decrease of processes number	Braissant et al. (2019)
BDL rats @ 6 weeks post-BDL Adult SD rats CSF ammonia^#^: 114.4 ± 12.2 µM	*Ex-vivo*: brain	^1^H and ^13^C MRS whole brain	↑ Gln, Glu ↓ mIns ↑Sum of all measured main brain organic osmolytes (Gln + Glu + mIns + Tau) ↑ de novo synthesis of Lac and Gln	Bosoi et al. (2014)
BDL rats @ 4 weeks post-BDL Plasma ammonia*: 186 ± 20 µM	*Ex-vivo*: brain	^1^H MRS	No change in brain Gln in BDL rats ↓ brain mIns in BDL rats	Davies et al. (2009)
BDL rats @ 6 weeks post-BDL	*In-vivo*: brain ^1^H MRS and DW-MRS experiments	^1^H MRS: 14.1T, STEAM, TE = 3 ms, cerebellum 6.5 × 4 × 3.6mm^3^ DW-MRS: 14.1T, STE-LASER, TE = 33 ms, cerebellum 6.5 × 4 × 3.6 mm^3^, bval: 0–15 ms/µm^2^, Td = 63ms	↑ Gln in BDL rats ↑ metabolites ADC, significant for Glu and Tau in BDL rats	Mosso et al. (2021)
Young BDL rats @ 4 and 6 weeks post-BDL (operated 21 days after birth)	*In-vivo*: brain ^1^H MRS, DW-MRI and DW-MRS experiments	^1^H MRS: 14.1T, STEAM, TE = 3 ms, cerebellum 6.5 × 4 × 3.6mm^3^ DW-MRS: 14.1T, STE-LASER, TE = 33 ms, cerebellum 6.5 × 4 × 3.6 mm^3^, bval: 0–15 ms/µm^2^, Td = 63 ms DW-MRI: 14.1T, spin echo EPI, TE = 29 ms, cerebellum 6.5 × 4 × 3.6 mm^3^ bval: 0–8 ms/µm^2^, Td = 12 ms	↑ Gln in BDL rats ↓ Osmolytes (mIns + Tau + tCho + tCr) in BDL rats ↑ metabolites ADC, significant for mIns, Tau, and tCr in BDL rats ↑ water MD in BDL rats	Mosso et al. (2022a)
BDL rats @ 6 weeks post-BDL	*In-vivo*: brain ^1^H MRS and PET experiments	^1^H MRS: 14.1T SPECIAL, TE = 2.8 ms 2 × 2.8 × 2 mm^3^ - hippocampus and 2.5 × 2.5 × 2.5 mm^3^ cerebellum ^18^F-FDG PET on the entire brain, co-registered with MRI and MRS results	Hippocampus: ↑ Gln in BDL rats ↓ Osmolytes (mIns + Tau + tCho + tCr) in BDL rats ↓ Glu, Asc in BDL rats Cerebellum: ↑ Gln in BDL rats ↓ Tau, Glu, GABA in BDL rats PET: 2-fold lower glucose cerebral metabolic rate (CMR) in BDL rats	Mosso et al. (2022b)

*Ammonia measured before sacrifice, # Values approximation – estimation from graphs

As mentioned before, HE is a multifactorial disease with different mechanisms acting together inducing neurological implications. As such, it has been shown in animal models that ammonia, OS, and inflammation all play a role in the pathogenesis of type C HE (Pierzchala et al. 2022; Wright et al. 2007a) (Table 4), as well as associated brain edema (cortex) (Bosoi et al. 2012). Treatment with allopurinol (an inhibitor of the oxidant enzyme xanthine oxidase (XO), an OS marker) reduced OS while normalizing water content in male rats (Bosoi et al. 2012), whilst treatment with probiotics reduced brain Gln concentrations (Rackayova et al. 2021). Furthermore, endotoxemia has been shown to impact brain edema in BDL rats following an increased inflammatory response (Wright et al. 2007a). Elevated ammonia, OS, and simultaneous inflammation induced ribonucleic acid (RNA) oxidation (8-Oxo-2’-deoxyguanosine (Oxo-8-dG) accumulation) in primary astrocytes cultures (Giorgio et al. 2020; Gorg et al. 2008; Korkmaz 2018; Kruczek et al. 2011; Schliess et al. 2009) and in the BDL rat model of type C HE, with dominating cytoplasmic localization indicating interplay of cytosolic and mitochondrial nucleic acids with hydroxyl radical (${\text{HO}}^{\cdot}$)(Pierzchala et al. 2022). RNA oxidation alters translational machinery and gene expression, revealing a link between ammonia-induced OS (Pierzchala et al. 2022) and cognitive impairment via altered protein synthesis (Nunomura et al. 2017) and neurotransmission (Kumar et al. 2018). EM of BDL cortical samples revealed partially collapsed microvessels, as well as minimal water accumulation in the astrocytic endfeet and perivascular tissue (Wright et al. 2007a). Enlarged astrocytic endfeet, perivascular edema, and collapsed microvessels were also evident in BDL rats following an acute inflammatory response (Wright et al. 2007a). Several brain areas from BDL rats showed enlarged mitochondria with ruptured membranes, unfolded cristae, and a non-dense matrix (Dhanda et al. 2018). These morphological alterations have been shown to contribute to the reported impaired ATP synthesis and amplify mitochondrial OS (Dhanda et al. 2018).

### Human type C HE

The main findings using *in-vivo* MRI and MRS techniques in patients with HE have been previously discussed (Cudalbu and Taylor-Robinson 2019). Volumetric MRI results have highlighted a decrease in brain volume in type C HE, mainly in gray matter , with functionally well-compensated patients with cirrhosis showing no brain volume changes. The changes in brain volume measured in chronic HE were mainly associated with brain atrophy (Bosoi and Rose 2013). A few absolute brain water content measurements have also been done in type C HE, showing a small increase in brain water content (around 1%) (Shah et al. 2008; Winterdahl et al. 2019) with other studies showing no significant increases in patients with covert HE (minimal HE or HE grade 1) (Oeltzschner et al. 2016). Magnetization transfer (MT) MR experiments highlighted a small decrease in magnetization transfer ratio in type C HE, which was consistent among studies and was interpreted as low-grade astrocytic/cerebral edema. This might also be linked to alterations in membrane permeability and cytoplasmic structure and to subsequent shifts in the distribution of macromolecules and intracellular water, with subtle alterations in intracellular and extracellular edema. Some studies where DW-MRI experiments were performed showed a mild increase in ADC in patients with cirrhosis, even with minimal HE . The overall agreement of all these measurements seems to be linked to an increase in water content, however additional studies are required to unravel the cellular origin which remains controversial. Future experiments using adapted biophysical models would potentially help elucidating these questions.

Based on the data published to date, the small change in water content in patients with type C HE are consistent and in line with the results obtained in animal studies. These results seem to highlight that in addition to brain edema other mechanisms are involved in type C HE, however further experiments are required.

^1^HMRS studies in patients with type C HE highlighted stronger changes in brain metabolites (Glx (Glu + Gln)/Cr, mIns/Cr, and tCho/Cr) in overt HE patients, while in minimal HE, the decrease in mIns/Cr was observed more often than an increase in Glx/Cr. In functionally well-compensated liver cirrhosis patients, no significant changes were measured (Cudalbu et al. 2023). Overall, these studies have been performed at lower magnetic fields, raising the questions as to whether metabolite changes occur in well-compensated liver disease patients, or if these changes are very small, and thus not detectable at lower magnetic fields.

## New approaches to evaluate brain edema and/or extra- and intracellular water content and microstructural changes

By virtue of the perivascular location of astrocytes and their high expression of water transporting membrane proteins, water is believed to preferentially enter via astrocyte membranes (Papadopoulos et al. 2004). This renders astrocytes more susceptible to swelling than neurons, especially when their adaptive capacity for volume regulation is exhausted (Bemeur et al. 2016; Bosoi and Rose 2013; Papadopoulos et al. 2004; Thrane et al. 2015). Morphological features of astrocytes, such as fine processes make up a large proportion of the cell volume and are in close contact with synapses and other components of brain parenchyma such as the vasculature (Wilhelmsson et al. 2006). Antibodies against GFAP, the most frequently used astrocyte marker (Eng et al. 2000), reveal the cytoskeletal structure but do not label all portions of the astrocyte. GFAP is entirely absent from the finely branching processes and is often not detectably present in the cell body. Consequently, GFAP immunohistochemistry can markedly underestimate the extent of astrocyte branching and territory, especially when performed in 2D. While other molecular markers have been used for immunohistochemical identification of astrocytes (e.g. GS (Norenberg 1979)), these molecules are also expressed by other cells (Sofroniew and Vinters 2010).

Astrocytes dynamically alter their volume to regulate physiological brain function, such as during sleep (O’Donnell et al. 2015) or extracellular potassium buffering (Florence et al. 2012). It is therefore essential to consider the importance of studying astrocytes in live tissue (*in-vivo* or *ex-vivo*) when investigating their role in brain edema. Two indirect parameters of astrocyte morphology have been established, which, while not fully resolving local geometry, provide statistical measures of astrocyte morphology (Minge et al. 2021). These are the fraction of tissue volume that astrocytes occupy and the density of resolvable astrocytic processes. They are relatively straightforward to obtain both *in-vivo* and in acute brain slices by impaling and iontophoretically injecting astrocytes with a fluorescent dye through a craniotomy (Wilhelmsson et al. 2004). Alexa Fluor 594 has often been chosen for this method as it provides 100% rapid staining of thin processes and even penetrates gap junctions (Savtchenko et al. 2019). Using two-photon excitation fluorescence microscopy, a widely available technique, z-stack of images can be collected containing the entire visible astrocyte structure, which is then analysed in a 3D format (Savtchenko et al. 2019). More powerful scanners that can rapidly scan many focal planes have also been combined with advanced genetic tools for monitoring Ca^2+^ gradients with high sensitivity, which allow for 3D Ca^2+^ imaging of a whole astrocyte evaluating its activity (Bindocci et al. 2017; Savtchouk et al. 2018). This method is highly complementary to morphological assessments but very challenging to analyse (Savtchouk et al. 2018). Furthermore, rodents can be habituated to be head-restraint so that they are awake during imaging, overcoming the need for general anaesthesia (Shih et al. 2012), but increasing further the complexity of data processing especially with Ca^2+^ imaging (Savtchouk et al. 2018). These type of imaging procedures require a chronic cranial window preparation, which is technically challenging, as well as imaging training. However, once mastered, they can be conducted without inducing astrogliosis (Hefendehl et al. 2011), and can also be applied to other cell types.

The definition of cytotoxic edema (isolated fluid shift from the interstitial to the intracellular compartment) might be controversial, as cytotoxic swelling would plausibly always be accompanied by some degree of net brain edema through other mechanisms, such as osmotic gradient across the BBB. Therefore, extracellular space volume measurements in addition to the morphological assessment of astrocytes are essential for the investigation of brain edema. There are several techniques available for the investigation of brain extracellular space volume (reviewed in (Nicholson and Hrabetova 2017; Soria et al. 2020; Sykova and Nicholson 2008)), which are mostly invasive (or at an experimental stage) and therefore mainly applied in rodent studies. Real-time iontophoresis (RTI) is the current gold-standard method for experimentally addressing extracellular space properties (volume and tortuosity), by measuring diffusional spread of a molecule, usually tetramethylammonium cation (TMA^+^) in ECS (Nicholson and Phillips 1981). TMA^+^ is applied iontophoretically *via* a glass capillary into the tissue, and time-dependent changes in concentration of TMA^+^ are detected within a known distance (typically 100–150 μm) by a TMA^+^ ion-selective microelectrode. Studies have reported adapted versions of the standard experimental procedure, where the use of fluorescent probes (e.g., Alexa488 and Alexan568) in both electrodes are included (Xie et al. 2013). This allows for their distance to be determined more accurately with fluorescence microscopy. A smaller extracellular space results in reduced TMA^+^ dilution, reflected by higher levels of detected TMA^+^ and vice versa.

The advantage of the RTI method is that it can be performed in brain slices, as well as *in-vivo*, and therefore be combined with the proposed cell morphological assessments. Due to the diffusion properties of TMA+, repeated time-lapse measurements (~ 5 min apart) can be obtained. This technique has also recently been used in awake animals (Xie et al. 2013), eliminating the effects of general anaesthesia, which have been shown to impair glymphatic system clearance and reduce extracellular space volume (Gakuba et al. 2018). On the other hand, the disadvantage associated with this method is that it does not visualize ECS structure and has very low spatial resolution relative to ECS structural geometries. Interpolation and averaging of tissue properties across the given distance are therefore required. Furthermore, due to the calibration being performed in aqueous agar, which represents an unhindered, homogeneous diffusion (Odackal et al. 2017) in regions with anisotropic diffusion, the ECS volume fraction has to be re-calculated in a different way (Vorisek and Sykova 1997). Combining this technique with complementary methods of microscopy, such as super-resolution shadow imaging (SUSHI), can eliminate many of the above limitations and provide more accurate and comprehensive analysis of the ECS volume. SUSHI is based on labelling of the interstitial fluid with a freely diffusible hydrophilic fluorophore that renders cellular structures visible as shadows. As the interstitial compartment is identical to the ECS in structural geometry, the ECS meshwork becomes directly visible allowing for its geometrical analysis (Soria et al. 2020; Tonnesen et al. 2018).

In addition to these techniques, DW-MRI and DW-MRS have been shown to be useful in HE due to their ability to characterize the microstructure of the brain *in-vivo* and non-invasively using water and metabolites complementary information. The estimated microstructure parameters resulting from these diffusion measures could extremely well complement the measurements described above (Fig. 2).

## Conclusion

The role of brain edema as a central event in HE has been questioned in the past, especially in type C HE where changes in water content are smaller, variable and sometimes controversial. It is now highly accepted that type A and type C HE are biologically and clinically different, leading to different manifestations of brain edema and that HE is a multifactorial disease where brain edema might not play a sole central role. The present review describes the main published *in-vitro, ex-vivo/in-vivo* findings using cell cultures, animal models and humans with HE, highlighting the presence of brain edema in HE. The majority of the *in-vitro* studies published till now focused on astrocytes and astrocytic swelling while other cell types such as the endothelial cells, neurons, microglia and pericytes in the brain are involved in the pathogenesis of HE. Additional studies should therefore focus on all cell types and asses their morphological changes using physiological ammonia concentrations relevant for type A and C HE. The water content changes reported in animal models of type C HE are small, variable but similar with what was measured in humans using absolute water measurements with MRI. The increase in brain Gln and decrease of the main osmolytes was consistent between animal models and human studies with type C HE, highlighting the presence of a volume regulatory response. In this context, methods with increased specificity, resolution and precision are required to assess absolute brain water changes together with cellular changes (both the extracellular space and cell volume/morphology) in HE. The usage of a multimodal approach combining *in-vivo* with *ex-vivo* experiments and allowing to monitor the progression of the disease longitudinally, could be instrumental in elucidating the central role of brain edema as a cause or consequence of HE and its implication in the development of the neurological alterations linked to HE.

## Data Availability

Not applicable.

## Abbreviations

ALF: Acute liver failure
ACLF: Acute on chronic liver failure
ADC: Apparent diffusion coefficient
BBB: Blood brain barrier
BDL: Bile-duct ligation
CBF: Cerebral blood flow
CLD: Chronic liver disease
CNS: Central nervous system
Cr: Creatine
CSF: Cerebrospinal fluid
DW-MRI: Diffusion-weighted MRI
DW-MRS: Diffusion-weighted MRS
ECS: Extracellular space
Gln: Glutamine
Glu: Glutamate
GM: Gray matter
GS: Glutamine synthetase
HE: Hepatic Encephalopathy
$${\text{HO}}^{\cdot}$$: Hydroxyl radical
ICP: Intracranial pressure
mIns: Myo-inositol
MRI: Magnetic resonance imaging
MRS: Magnetic resonance spectroscopy
MT: Magnetization transfer
NAA: N-acetylaspartate
NF-κB: Nuclear factor-κ B
IFN-γ: Interferon-γ
IL-1β: Interleukin 1β
IL-6: Interleukin 6
OS: Oxidative stress
$${\text{O}}_{2}^{\cdot -}$$: Superoxide anion
Oxo-8-dG: 8-Oxo-2’-deoxyguanosine
PCA: Portocaval anastomosis
Tau: Taurine
tCho: Total choline
TNF-α: Tumor necrosis factor-α
WM: White matter
